# Disease burden and related risk factors of esophageal cancer in China and globally from 1990 to 2021, with forecast to 2035: An analysis and comparison

**DOI:** 10.18332/tid/191389

**Published:** 2024-08-01

**Authors:** Mimi Wang, Huiwen Miao

**Affiliations:** 1 Science and Education Information Section, Hangzhou Center for Disease Control and Prevention, Hangzhou, China; 2 Department of Thoracic Surgery, The First Affiliated Hospital of Zhejiang, University School of Medicine, Hangzhou, China

**Keywords:** esophageal cancer, mortality rate, DALYs, trend

## Abstract

**INTRODUCTION:**

In this study we estimate the burden of esophageal cancer (EC) in China and globally from 1990 to 2021, with a forecast to 2035, using Global Burden of Disease (GBD) data. We also analyze the related risk factors to investigate burden trends.

**METHODS:**

Mortality, disability-adjusted life years (DALYs), crude rates, and age-standardized rates of EC were analyzed in China and globally from 1990 to 2035, utilizing GBD open data as a secondary dataset analysis of GBD data. Temporal change trends of EC risk factors were analyzed from 1990 to 2021. Joinpoint regression determined average annual percentage change (AAPC) of age-standardized rates. Descriptive analysis compared mortality and DALYs by age groups. Bayesian age-period-cohort (BAPC) predicted age-standardized mortality and DALYs rates for the next 14 years.

**RESULTS:**

The ASMR and ASDR fluctuations in EC were significant in China, showing an overall downward trend. Globally, although there was also a downward trend, the fluctuations were relatively mild. The number of deaths and DALYs related to EC in China and globally showed a significant upward trend. Age-specific burden trends in China for EC indicated that the age group with the peak number of EC deaths shifted to the 70–74 years age group in 2021, while DALYs peaked in the 65–69 years age group. The crude mortality rate (CMR) peaked consistently in 1990 and 2021, both within the 90–94 years age range, while the crude DALY rate (CDR) shifted to the 85–89 years age group. Overall, the burden of EC deaths and DALYs in the population aged <40 years was relatively low, increasing rapidly after the age of 40 years, reaching a peak and gradually declining, and reaching a lower level after the age of 85 years. The predictive results of the BAPC model indicated that over the next 14 years, both ASMR and ASDR for EC in China and globally would show a slight overall increase. The GBD 2021 study identified smoking, high alcohol use, chewing tobacco, and diet low in vegetables as the main risk factors affecting EC mortality rate and DALYs. Among these, smoking and alcohol use were the most significant risk factors, with a higher impact on EC in China compared to the global level. From 1990 to 2021, the overall changes in ASMR and ASDR indicate a decreasing trend in the impact of these four risk factors on EC mortality rate and DALYs.

**CONCLUSIONS:**

The burden of EC is expected to steadily increase in China and globally until 2035, posing a significant challenge. Targeted prevention and control policies, such as calling on people to quit smoking and reduce alcohol use, may help curb this upward trend.

## INTRODUCTION

Esophageal cancer (EC), encompassing both esophageal squamous cell carcinoma (ESCC) and esophageal adenocarcinoma (EAC), ranks seventh in terms of global cancer incidence and sixth in terms of mortality. It presents a formidable challenge to human health, especially among vulnerable populations^1^. China has been a high-incidence country for EC, with over 50% of global cases, with ESCC being the main type of EC^2,3^. Typically, ESCC originates from the squamous epithelium of the esophageal mucosa. When the mucosa is exposed to carcinogens or mechanical damage, epithelial cells undergo abnormal proliferation, ultimately developing into invasive cancer^4,5^. According to the latest release of global cancer burden data in 2020 by the International Agency for Research on Cancer, a division of the World Health Organization, EC ranks fourth among cancer deaths in China^2^. Therefore, the burden of EC in China is significant and urgently requires a comprehensive strategy to address the worsening situation.

China has been a beneficiary of international cooperation and support in the field of EC disease control since the 1970s. Simultaneously, as China’s socioeconomic conditions continue to improve, remarkable progress has been made in the prevention, diagnosis, epidemiology, treatment, and research of EC^6^. However, due to distant metastasis in about half of the patients with EC, combined with its heterogeneity, EC has inherent resistance to chemotherapy^7-9^. Therefore, although China has made the latest advancement in treating metastatic EC, the prognosis for EC patients remains relatively poor, having a 5-year survival rate of about 15–25%^10,11^. Given the increasingly heavy burden of EC, researchers have found that many etiological or risk factors contribute to the development of EC, such as smoking and excessive alcohol use^12^. Regarding smoking, the World Health Organization (WHO) has developed effective measures in the Framework Convention on Tobacco Control, such as demand-reduction strategies, advertising regulations, and tobacco taxation^13,14^. China, the largest country in East Asia regarding land area and population, has implemented targeted Healthy China 2030 tobacco control strategies to reduce smoking rates^15^. Additionally, dietary factors such as reduced intake of vegetables and dietary vitamins^16^, obesity and high body mass index (BMI), and gastroesophageal reflux disease^17^ can also promote malignant progression of EC. Therefore, early detection, treatment, management, and prevention of related risk factors for EC are crucial. A comprehensive understanding of the latest epidemiological characteristics of EC in China and the revelation of burden-related risk factors can promote the development of more scientific and rational policies.

Currently, research on the epidemiology of EC in China has strong regional limitations. For example, Zhang et al.^18^ studied EC in Henan Province from 2010 to 2018, while Wang et al.^19^ evaluated the incidence and mortality trends of EC in Huai’an District, Huai’an City, Jiangsu Province from 1990 to 2016. Previous studies cannot fully describe the recent EC burden in China. Recently, a study using the Global Burden of Disease (GBD) database evaluated the trend of EC burden in China employing relevant indicators such as incidence, mortality, and disability-adjusted life years (DALYs) from 1990 to 2019^20^. However, the above study described the burden of EC without in-depth exploration of risk factors and future trends of EC in China, insufficiently covering the epidemiology in China. To fill this gap, in this study we analyze the death and DALYs burden data of EC in China and on a global scale over the past 30 years using the GBD 2021 database. For 1990 to 2021, we conducted an analysis of the temporal change trends of risk factors associated with EC in both China and the global context. Furthermore, we employed Bayesian age-period-cohort (BAPC) model to predict the future burden of EC for the upcoming 14 years.

## METHODS

### Data source

Our data were from GBD 2021 (https://vizhub.healthdata.org/gbd-results/). This is a secondary dataset analysis of the GBD study. Specifically, we downloaded the following data for subsequent analysis: 1) annual age-specific data on EC from 1990 to 2021 in China and globally, including the number of deaths and DALYs and their corresponding crude rates, namely crude mortality rate (CMR) and crude DALYs rate (CDR), as well as their corresponding age-standardized rates (ASR), including age-standardized mortality rate (ASMR) and age-standardized DALYs rate (ASDR); 2) population data by age groups for China and the world from 1990 to 2021; and 3) ASR data on related risk factors attributed to EC in China and globally in 1990 and 2021. The data collection and access time for this study was 26 June 2024. Since we used publicly available databases, this study did not require ethical approval or informed consent.

### Statistical analysis

*Descriptive analysis*

This study employed descriptive analysis to examine the temporal and age-related trends of EC burden in China and on a global scale. Furthermore, it analyzed the temporal change trend of risk factors associated with EC from 1990 to 2021. All data utilized in the descriptive analysis were subjected to statistical analysis utilizing R software (version 4.2.1).

*Joinpoint regression analysis*

A joinpoint regression model, consisting of linear statistical models, was employed to assess the temporal trend of disease burden caused by EC. The model estimated the changes in death and DALY rates utilizing the least squares method, avoiding the subjectivity of trend analysis based on linear trends. The sum of squares of residuals between the estimated values and actual values was calculated to obtain the inflection points of the moving trend. Joinpoint software (version 4.9.1.0; National Cancer Institute, Rockville, Maryland, USA) was applied to build this model. We also calculated the average annual percentage change (AAPC) and investigated whether the fluctuation trends of different sections were statistically significant by comparing AAPC with zero. Statistical significance was set at p<0.05^21,22^.

*BAPC model*

The BAPC model is a complex statistical tool that combines prior information on unknown parameters with sample information to estimate the posterior distribution and infer these unknown parameters^23^. It has been shown to have higher accuracy in predicting disease burden. Therefore, we used it in the integrated nested Laplace approximation (INLA) using the R packages BAPC and INLA to predict the mortality and DALYs burden of EC in China and globally from 2022 to 2035. All data analyses were conducted using the open-source software R (version 4.2.1).

## RESULTS

### Trends in the burden of EC in China and globally, 1990–2021

The changes in the number of EC mortality, ASMR, DALYs, and ASDR (per 100000 population) in China and globally in 1990 and 2021 are presented in Table 1. According to the data, in 2021, EC in China resulted in 296443 deaths (95% UI: 236648-362831), representing an increase of 40.61% compared to 1990. Additionally, the number of DALYs attributed to EC in China increased by 17.88% compared to 1990. On a global scale, the number of deaths caused by EC in 2021 was 538602 (95% UI: 475944–603406), indicating a 51.18% increase compared to 1990. The number of DALYs attributed to EC globally increased by 33.28% compared to 1990. However, when considering the changes in EC ASMR and ASDR, in 2021, the ASMR in China decreased by 45.78% compared to 1990, and the ASDR decreased by 51.45%. Furthermore, in 2021, the global ASMR for EC decreased by 30.67% compared to 1990, and the ASDR decreased by 36.87%.

**Table 1:** Changes in esophageal cancer mortality, ASMR, DALYs, ASDR (per 100000) in China and globally in 1990 and 2021

*Measure*	*China*			*Global*		
	*1990 n (95% UI)*	*2021 n (95% UI)*	*Change %*	*1990 n (95% UI)*	*2021 n (95% UI)*	*Change %*
Mortality	210821 (176081–244587)	296443 (236648–362831)	40.61	356263 (319363–390154)	538602 (475944–603406)	51.18
ASMR	26.06 (21.77–30.1)	14.13 (11.36–17.18)	45.78	9.02 (8.11–9.87)	6.25 (5.53–7)	-30.67
DALYs	5852132 (4841614–6818927)	6898666 (5471181–8553366)	17.88	9753566 (8719319–10739561)	12999265 (11522861–14605268)	33.28
ASDR	653.31 (543.18–758.88)	317.18 (252.46–392.42)	-51.45	235.32 (210.52–258.68)	148.56 (131.71–166.82)	-36.87

UI: uncertainty interval. ASMR: age-standardized mortality rate. ASDR: age-standardized DALYs rate.

**Figure 1:**
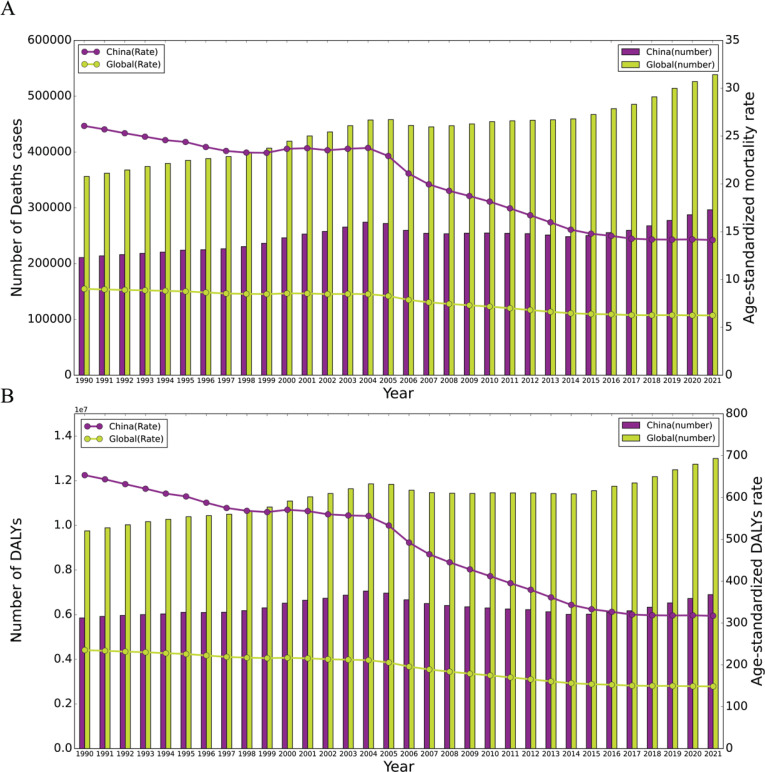
A) Trends in the number of deaths and ASMR for EC in China and globally from 1990 to 2021; B) Trends in the number of DALYs and ASDR for EC in China and globally from 1990 to 2021. The bar graphs represent the quantity, while the line graphs represent the age-standardized rates (ASR)

Figure 1 illustrates the trends in deaths and DALYs due to EC in China and on a global scale from 1990 to 2021. In China, ASMRs and ASDRs for EC showed significant fluctuations, exhibiting a decrease followed by an increase, reaching a peak in 2004, and then sharply decreasing again (Supplementary file Table S1). In contrast, at the global level, fluctuations in ASMR and ASDR for EC were relatively stable, illustrating an overall decreasing trend. Additionally, overall, both ASMR and ASDR for EC in China from 1990 to 2021 were consistently higher than the global levels. Regarding the number of deaths and DALYs due to EC, both China and the world showed an increasing trend.

### Joinpoint regression analysis

**Figure 2:**
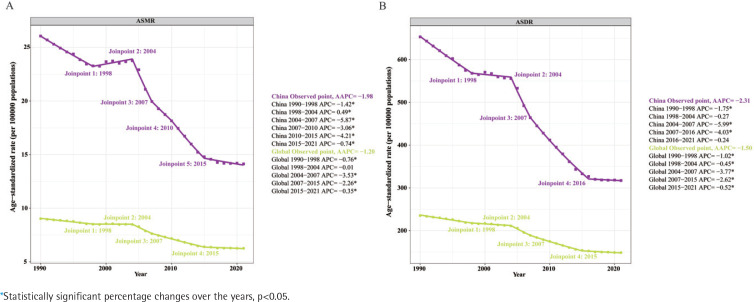
A) Joinpoint regression analysis of ASMR for EC in China and globally from 1990 to 2021; B) Joinpoint regression analysis of ASDR for EC in China and globally from 1990 to 2021

The APCs of ASMR and ASDR for EC in China and globally from 1990 to 2021, along with the corresponding data, are shown in Figure 2 and Supplementary file Table S2. The APC of ASMR for EC in China exhibited a significant initial decrease (1990–1998, APC= -1.42, p<0.05), followed by a significant increase (1998–2004, APC=0.49, p< 0.05), reaching a peak in 2004. Subsequently, from 2004 to 2021, there was an overall stable decline (Figure 2A). The AAPC of ASMR for EC in China from 1990 to 2021 was -1.98. The APC of ASDR for EC in China showed a similar trend to ASMR (Figure 2B), with an AAPC of -2.31 from 1990 to 2021. Additionally, the APCs of ASMR and ASDR for EC globally exhibited a similar pattern to China, with a slight initial decrease (ASMR: 1990–1998, APC= -0.76; ASDR: 1990–1998, APC= -1.02; p< 0.05), followed by a slight increase, reaching peaks in 2004, and then an overall stable decline from 2004 to 2021. The AAPC of ASMR for global EC from 1990 to 2021 was -1.20, and the AAPC of ASDR was -1.50.

### Trends in the burden of EC in different age groups in China and globally in 1990 and 2021

**Figure 3:**
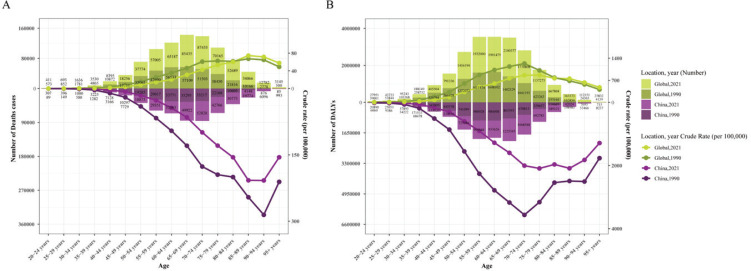
A) Number of deaths and crude mortality rate (CMR) for EC in different age groups in China and globally in 1990 and 2021; B) Number of DALYs and crude DALYs rate (CDR) for EC in different age groups in China and globally in 1990 and 2021. The bar graphs represent the quantity, while the line graphs represent the crude rates (CR)

Furthermore, we conducted further analysis to derive the burden trends of EC in different age groups in China, specifically comparing data from 1990 and 2021 (Figure 3, Supplementary file Table S3). As shown in Figure 3A, the highest number of EC deaths in China in 1990 occurred in the 65–69 years age group, while in 2021, this peak shifted to the 70–74 years age group. In terms of CMR, the peaks in both 1990 and 2021 were consistent and occurred in the 90–94 years age group (Figure 3A). As for the number of DALYs due to EC, the peak in 1990 occurred in the 60–64 years age group, while in 2019, the peak shifted to the 65–69 years age group (Figure 3B). Similarly, for CDR, the peak age group for EC in 1990 was 70–74 years, while in 2021, it was 85–89 years age group (Figure 3B). Overall, the burden of EC, measured by deaths and DALYs, was low in individuals aged <40 years and rapidly increased after the age of 40 years, reaching a peak and then gradually decreasing to a lower level after the age of 85 years.

### Forecasting ASMR and ASDR of EC from 2022 to 2035

**Figure 4:**
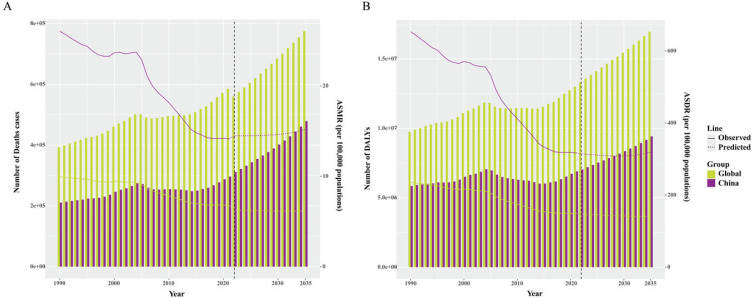
A) Forecasted number of deaths and ASMR for EC in China and globally over the next 15 years; B) Forecasted number of DALYs and ASDR for EC in China and globally over the next 15 years. The bar graphs represent the quantity, while the line graphs represent ASR

In this study, we utilized the GBD data from 1990 to 2021 and employed the BAPC model to project the trends in EC disease burden from 2022 to 2035. The results are presented in Figure 4 and Supplementary file Table S4. The forecasted outcomes indicated that over the next 14 years, both the number of deaths and DALYs attributed to EC would continue to increase in China and globally. The changes in ASMR and ASDR for EC showed a similar pattern, with an overall gradual and modest upward trend.

### Trends in EC-related risk factors from 1990 to 2021

**Figure 5:**
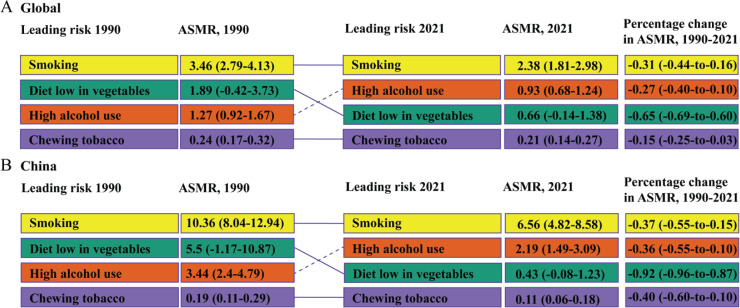
Detailed ranking of the main risk factors attributing to ASMR for EC in China and globally from 1990 to 2021. A) Global; B) China. Dashed lines indicate an increase in rank. Solid lines indicate a decrease in rank or no change. Data in parentheses represent the 95% uncertainty interval

**Figure 6:**
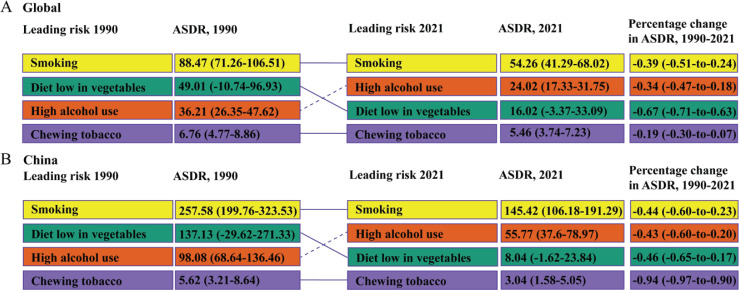
Detailed ranking of the main risk factors attributing to ASDR for EC in China and globally from 1990 to 2021. A) Global. B) China. Dashed lines indicate an increase in rank. Solid lines indicate a decrease in rank or no change. Data in parentheses represent the 95% uncertainty interval

Based on the GBD data for 2021, smoking, high alcohol use, chewing tobacco, and a diet low in vegetables were identified as the primary risk factors influencing EC mortality. The analysis of the risk factors affecting ASMR and ASDR for EC in China and globally, as shown in Figures 5–6, revealed that smoking was the most significant risk factor impacting EC mortality and DALYs, and its risk effect on EC mortality and DALYs in China was higher than the global level. Furthermore, in 1990, the second, third, and fourth leading risk factors affecting EC mortality and DALYs in China and globally were diet low in vegetables, high alcohol use, and chewing tobacco, respectively. However, by 2021, high alcohol use surpassed the diet low in vegetables to become the second leading risk factor. Lastly, when considering the overall changes in ASMR and ASDR from 1990 to 2021, the impact of these four risk factors – smoking, alcohol use, chewing tobacco, and diet low in vegetables – on EC mortality and DALYs exhibited a decreasing trend.

## DISCUSSION

To the best of our knowledge, this is the first comparative analysis conducted on GBD 2021 data on the burden of EC and its associated risk factors in both China and the global context. We found that the number of EC deaths and DALYs in China and globally has shown a significant increasing trend, while the ASMR and AMDR have overall shown a decreasing trend, which was mainly related to the rapid aging of the population. The trend of EC burden in different age groups in China showed that the age group with the peak number of EC deaths and DALYs in 2021 was older compared to 1990. The CMR and CDR of EC in China and globally have both shown a small upward trend, and the elderly were the main population affected by EC death and disability, with their mortality and disability rates increasing with age. The analysis of EC mortality and DALYs-related risk factors showed that smoking and alcohol use were the greatest risk factors affecting EC disease mortality and DALYs in China and the global context, with a higher risk of impact on China than on a global scale. BAPC model forecasted that the ASMR and ASDR of EC in China and the global context would show an overall small upward trend in the next 14 years, and the burden of EC would keep increasing to 2035. Therefore, it is crucial to formulate policies for diagnosis, treatment, and management based on a thorough understanding of the epidemiology of EC.

The ASMR and ASDR burden of EC in China and globally in this study showed an upward trend followed by a downward trend, which was similar to the results reported in previous related studies^24^. Previous studies have shown that the trend of EC ASMR from 2000 to 2011 is consistent with the age-standardized incidence rate (ASIR) of EC, and the decrease in ASMR may be strongly linked with the decrease in EC ASIR^25^. Changes in ASMR reflect differences in exposure to risk factors, socioeconomic environment, lifestyle, and access to cancer care and screening^26,27^. Since 2006, China has strengthened measures to promote early screening, diagnosis, and treatment of EC, including the Early Diagnosis and Early Treatment Program in 2006^28^, the Anyang EC Cohort Study in 2006^29^, the Esophageal, Gastric, and Liver Cancer Screening Program in 2007^30^, and the Cancer Screening Program in Urban China in 2012^31^. The implementation of these EC prevention and control policies has identified a considerable number of pre-cancerous lesions and EC patients, prompting them to seek medical treatment early, inhibiting the progression of the disease to late-stage EC, and thus reducing the mortality and disability of EC. This may be an important reason for the decrease in the burden of EC since 2004 and the decrease in the trend higher than the global level. However, the BAPC model forecast in this study showed that the ASMR and ASDR of EC in China and globally will show an upward trend in the next 14 years, and the pressure of EC disease prevention and control still exists. It is necessary to strengthen targeted prevention and control measures and implement prevention, diagnosis, and treatment for specific populations on the basis of the current overall prevention and control policy, in the hope of curbing the upward trend of EC burden.

The study on risk factors for the burden of disease has found that smoking, alcohol use, diet low in vegetables, chewing tobacco, and high BMI are the main factors contributing to EC death and disability in China and globally^17,32,33^. Based on the GBD 2019 data in this study, smoking and alcohol use remain the most vital risk factors for EC in China and on a global scale. A study of 195 countries found that a significant proportion of the DALYs caused by EC is attributed to smoking and alcohol use^17^. A meta-analysis of smoking and EC risk in non-Hispanic White individuals found that smokers are almost twice as likely to develop EC as non-smokers, and the severity of the addiction is significantly associated with long-term outcomes in patients^17^. In addition, prior research has substantiated that consumption of fresh fruits and vegetables is associated with lower incidence of EC. Eating fresh vegetables and citrus fruits according to the principles of the Mediterranean diet can stem the occurrence of ESCC^16,34^, which may be related to the antioxidant properties inherent in fruits and vegetables that can mitigate oxidative stress and inflammation. In conclusion, when formulating health promotion strategies for EC, the risk factors of smoking, alcohol use, and diet low in vegetables should be considered, and individuals exposed to these risk factors should be given correct publicity and education and timely prevention measures. At the same time, in China, calling on the public to quit smoking, reduce alcohol use, and manage body weight should be an important part of the prevention strategy.

In our work, the age-specific death rate and DALYs of EC gradually increased with age, and the death rate and DALYs of EC in the population aged <40 years were at a lower level, but after 40 years, they rapidly increased, reaching a peak and then gradually decreasing to a lower level after the age of 85 years. The increase in the death rate and DALYs number after the age of 40 years may be due to the absence of typical endoscopic features in early EC, so most individuals are diagnosed in the middle and late stages when the prognosis is poor, the survival rate is low, and thus the death rate and DALYs are high^35,36^. The high death rate and DALYs in the elderly may be related to their physical health, related chronic diseases, and poor immunity. Therefore, it is advisable to prioritize individuals aged ≥40 years as a pivotal demographic for the prevention, early detection, and management of EC.

### Limitations

Although this study contributes to our understanding of trends in the EC burden and related risk factors in China and globally, some limitations exist. Firstly, the accuracy of GBD estimates is predominantly contingent upon the quality and quantity of the data employed. For example, in regions like Asia, South America, and Africa, the proportion of the population covered during cancer registration is low, which may lead to biased estimated data. Secondly, there is underreporting or underdiagnosis during cancer registration, especially in resource-limited developing countries, which may lead to an underestimation of the number of deaths and DALYs caused by EC globally. In addition, the EC diagnostic criteria for these data sources may differ and affect the accuracy of the estimates in the GBD database. Finally, the GBD data used here did not differentiate between the two major histological subtypes included in EC but instead provided overall aggregated statistics, which may limit the representation of the disease burden specific to individual EC subtypes.

## CONCLUSIONS

This is the first study to compare and analyze data on the burden of EC and related risk factors in China and globally from the 2021 GBD database. The study findings revealed a significant increase in the number of deaths and DALYs associated with EC in both China and the world, while ASMR and ASDR showed an overall decreasing trend. Smoking and alcohol use emerged as the primary risk factors for EC in China and globally, with China exhibiting a higher risk level. Over the next 14 years, a slight increase is projected in the ASMR and ASDR for EC in China and globally. Therefore, it is crucial to enhance prevention and control measures, particularly among high-risk populations such as smokers, alcohol consumers, and individuals with diet low in vegetables. Implementing effective strategies for prevention, early screening, diagnosis, and treatment, specifically targeted at these high-risk groups, is of paramount importance. Such efforts will help curb the rising burden of EC.

## Supplementary Material



## Data Availability

The data supporting this research can be found in the Global Burden of Disease Study 2019.
