# Supplementary material for: Disease burden and related risk factors of esophageal cancer in China and globally from 1990 to 2021, with forecast to 2035: An analysis and comparison
Source: Tob Induc Dis. 2024 Aug 1;22:10.18332/tid/191389. doi: 10.18332/tid/191389 (PMC11292605; doi:10.18332/tid/191389)
Supplement: Supplementary file 1 [file TID-22-140-s1.pdf]

measure	location	sex	age	cause	metric	year	val	upper	lower
Deaths	Global	Both	Age-standardized	Esophageal cancer	Rate	1992	8,91	9,73	8,07
Deaths	Global	Both	Age-standardized	Esophageal cancer	Rate	1997	8,54	9,13	7,75
Deaths	Global	Both	Age-standardized	Esophageal cancer	Rate	1994	8,81	9,50	7,97
Deaths	Global	Both	Age-standardized	Esophageal cancer	Rate	1990	9,02	9,87	8,11
Deaths	China	Both	Age-standardized	Esophageal cancer	Rate	1990	26,06	30,10	21,77
Deaths	China	Both	Age-standardized	Esophageal cancer	Rate	1992	25,30	29,07	21,36
Deaths	China	Both	Age-standardized	Esophageal cancer	Rate	1994	24,58	27,63	20,82
Deaths	China	Both	Age-standardized	Esophageal cancer	Rate	1997	23,45	26,12	19,99
Deaths	Global	Both	Age-standardized	Esophageal cancer	Rate	1998	8,50	9,06	7,57
Deaths	Global	Both	Age-standardized	Esophageal cancer	Rate	1995	8,76	9,37	7,82
Deaths	China	Both	Age-standardized	Esophageal cancer	Rate	1999	23,25	25,88	19,48
Deaths	China	Both	Age-standardized	Esophageal cancer	Rate	1998	23,27	25,88	19,38
Deaths	Global	Both	Age-standardized	Esophageal cancer	Rate	1991	8,96	9,77	8,09
Deaths	China	Both	Age-standardized	Esophageal cancer	Rate	1991	25,70	29,46	21,65
Deaths	China	Both	Age-standardized	Esophageal cancer	Rate	1995	24,38	27,15	20,32
Deaths	Global	Both	Age-standardized	Esophageal cancer	Rate	2003	8,50	9,06	7,64
Deaths	China	Both	Age-standardized	Esophageal cancer	Rate	2005	22,92	25,45	19,42
Deaths	China	Both	Age-standardized	Esophageal cancer	Rate	2003	23,67	26,17	19,84
Deaths	Global	Both	Age-standardized	Esophageal cancer	Rate	2000	8,55	9,21	7,69
Deaths	Global	Both	Age-standardized	Esophageal cancer	Rate	2001	8,54	9,18	7,66
Deaths	China	Both	Age-standardized	Esophageal cancer	Rate	2000	23,66	26,53	20,05
Deaths	China	Both	Age-standardized	Esophageal cancer	Rate	2004	23,76	26,41	20,05
Deaths	Global	Both	Age-standardized	Esophageal cancer	Rate	2006	7,87	8,41	7,11
Deaths	China	Both	Age-standardized	Esophageal cancer	Rate	2006	21,09	23,47	17,91
Deaths	Global	Both	Age-standardized	Esophageal cancer	Rate	1999	8,48	9,09	7,61
Deaths	China	Both	Age-standardized	Esophageal cancer	Rate	2001	23,73	26,71	20,07
Deaths	Global	Both	Age-standardized	Esophageal cancer	Rate	2004	8,48	9,09	7,61
Deaths	Global	Both	Age-standardized	Esophageal cancer	Rate	2008	7,45	7,94	6,86
Deaths	Global	Both	Age-standardized	Esophageal cancer	Rate	2005	8,28	8,83	7,49
Deaths	China	Both	Age-standardized	Esophageal cancer	Rate	2010	18,15	20,37	15,56

Deaths	China	Both	Age-standardized	Esophageal cancer	Rate	2008	19,28	21,39	16,87
Deaths	Global	Both	Age-standardized	Esophageal cancer	Rate	2007	7,62	8,15	6,88
Deaths	China	Both	Age-standardized	Esophageal cancer	Rate	2007	19,95	22,26	16,95
Deaths	Global	Both	Age-standardized	Esophageal cancer	Rate	2011	7,00	7,51	6,46
Deaths	China	Both	Age-standardized	Esophageal cancer	Rate	2011	17,44	19,45	15,28
Deaths	China	Both	Age-standardized	Esophageal cancer	Rate	2009	18,74	20,74	16,22
Deaths	Global	Both	Age-standardized	Esophageal cancer	Rate	2013	6,63	7,15	6,11
Deaths	Global	Both	Age-standardized	Esophageal cancer	Rate	2009	7,31	7,78	6,69
Deaths	China	Both	Age-standardized	Esophageal cancer	Rate	2013	15,98	17,87	14,04
Deaths	Global	Both	Age-standardized	Esophageal cancer	Rate	2015	6,40	6,94	5,87
Deaths	Global	Both	Age-standardized	Esophageal cancer	Rate	2012	6,81	7,37	6,26
Deaths	Global	Both	Age-standardized	Esophageal cancer	Rate	2010	7,18	7,69	6,55
Deaths	China	Both	Age-standardized	Esophageal cancer	Rate	2015	14,79	16,92	12,75
Deaths	China	Both	Age-standardized	Esophageal cancer	Rate	2012	16,72	18,96	14,61
Deaths	Global	Both	Age-standardized	Esophageal cancer	Rate	2014	6,47	7,03	5,91
Deaths	China	Both	Age-standardized	Esophageal cancer	Rate	2017	14,27	17,04	11,97
Deaths	Global	Both	Age-standardized	Esophageal cancer	Rate	2018	6,27	6,98	5,62
Deaths	Global	Both	Age-standardized	Esophageal cancer	Rate	2016	6,36	6,96	5,75
Deaths	China	Both	Age-standardized	Esophageal cancer	Rate	2016	14,57	16,90	12,33
Deaths	China	Both	Age-standardized	Esophageal cancer	Rate	2014	15,22	17,41	13,18
Deaths	Global	Both	Age-standardized	Esophageal cancer	Rate	2019	6,28	7,01	5,60
Deaths	China	Both	Age-standardized	Esophageal cancer	Rate	2018	14,19	16,92	11,58
Deaths	Global	Both	Age-standardized	Esophageal cancer	Rate	2017	6,28	6,98	5,68
Deaths	Global	Both	Age-standardized	Esophageal cancer	Rate	1993	8,87	9,62	7,99
Deaths	Global	Both	Age-standardized	Esophageal cancer	Rate	1996	8,65	9,25	7,76
Deaths	China	Both	Age-standardized	Esophageal cancer	Rate	2019	14,18	16,96	11,68
Deaths	Global	Both	Age-standardized	Esophageal cancer	Rate	2021	6,25	7,00	5,53
Deaths	Global	Both	Age-standardized	Esophageal cancer	Rate	2002	8,48	9,09	7,58
Deaths	China	Both	Age-standardized	Esophageal cancer	Rate	2021	14,13	17,18	11,36
Deaths	Global	Both	Age-standardized	Esophageal cancer	Rate	2020	6,26	6,99	5,54
Deaths	China	Both	Age-standardized	Esophageal cancer	Rate	1993	24,94	28,35	21,03

Deaths	China	Both	Age-standardized	Esophageal cancer	Rate	1996	23,85	26,66	20,05
Deaths	China	Both	Age-standardized	Esophageal cancer	Rate	2002	23,52	26,14	19,77
Deaths	China	Both	Age-standardized	Esophageal cancer	Rate	2020	14,20	16,98	11,47
DALYs (Disability-Adjusted Life Years)	Global	Both	Age-standardized	Esophageal cancer	Rate	1990	235,32	258,68	210,52
DALYs (Disability-Adjusted Life Years)	China	Both	Age-standardized	Esophageal cancer	Rate	1991	643,40	741,57	543,36
DALYs (Disability-Adjusted Life Years)	China	Both	Age-standardized	Esophageal cancer	Rate	1990	653,31	758,88	543,18
DALYs (Disability-Adjusted Life Years)	Global	Both	Age-standardized	Esophageal cancer	Rate	1994	227,87	246,56	207,74
DALYs (Disability-Adjusted Life Years)	China	Both	Age-standardized	Esophageal cancer	Rate	1996	587,46	657,60	497,26
DALYs (Disability-Adjusted Life Years)	China	Both	Age-standardized	Esophageal cancer	Rate	1995	602,43	678,34	506,39
DALYs (Disability-Adjusted Life Years)	Global	Both	Age-standardized	Esophageal cancer	Rate	1995	225,92	242,29	202,79
DALYs (Disability-Adjusted Life Years)	China	Both	Age-standardized	Esophageal cancer	Rate	1993	620,90	712,28	530,45
DALYs (Disability-Adjusted Life Years)	Global	Both	Age-standardized	Esophageal cancer	Rate	1993	230,10	250,33	207,81
DALYs (Disability-Adjusted Life Years)	China	Both	Age-standardized	Esophageal cancer	Rate	1994	609,29	690,61	518,10
DALYs (Disability-Adjusted Life Years)	China	Both	Age-standardized	Esophageal cancer	Rate	1992	631,83	731,62	529,96
DALYs (Disability-Adjusted Life Years)	China	Both	Age-standardized	Esophageal cancer	Rate	1997	574,86	642,74	494,32
DALYs (Disability-Adjusted Life Years)	China	Both	Age-standardized	Esophageal cancer	Rate	1998	568,10	633,71	480,80
DALYs (Disability-Adjusted Life Years)	China	Both	Age-standardized	Esophageal cancer	Rate	2002	559,74	623,48	476,31
DALYs (Disability-Adjusted Life Years)	Global	Both	Age-standardized	Esophageal cancer	Rate	1991	233,54	255,39	210,73
DALYs (Disability-Adjusted Life Years)	Global	Both	Age-standardized	Esophageal cancer	Rate	1992	231,64	254,20	208,94
DALYs (Disability-Adjusted Life Years)	China	Both	Age-standardized	Esophageal cancer	Rate	2001	567,50	640,84	484,34
DALYs (Disability-Adjusted Life Years)	China	Both	Age-standardized	Esophageal cancer	Rate	2003	557,10	619,89	473,95
DALYs (Disability-Adjusted Life Years)	China	Both	Age-standardized	Esophageal cancer	Rate	2000	570,60	639,60	489,10
DALYs (Disability-Adjusted Life Years)	China	Both	Age-standardized	Esophageal cancer	Rate	2004	555,75	619,29	478,40
DALYs (Disability-Adjusted Life Years)	China	Both	Age-standardized	Esophageal cancer	Rate	1999	565,11	629,75	479,01
DALYs (Disability-Adjusted Life Years)	Global	Both	Age-standardized	Esophageal cancer	Rate	1996	222,40	238,00	200,61
DALYs (Disability-Adjusted Life Years)	Global	Both	Age-standardized	Esophageal cancer	Rate	1999	216,14	230,75	193,73
DALYs (Disability-Adjusted Life Years)	Global	Both	Age-standardized	Esophageal cancer	Rate	2001	215,61	232,26	195,16
DALYs (Disability-Adjusted Life Years)	China	Both	Age-standardized	Esophageal cancer	Rate	2009	428,18	473,39	376,91
DALYs (Disability-Adjusted Life Years)	China	Both	Age-standardized	Esophageal cancer	Rate	2010	411,94	464,67	356,37
DALYs (Disability-Adjusted Life Years)	Global	Both	Age-standardized	Esophageal cancer	Rate	1998	217,20	232,00	195,02
DALYs (Disability-Adjusted Life Years)	China	Both	Age-standardized	Esophageal cancer	Rate	2007	464,22	515,24	403,50

DALYs (Disability-Adjusted Life Years)	Global	Both	Age-standardized	Esophageal cancer	Rate	2000	216,89	233,37	196,26
DALYs (Disability-Adjusted Life Years)	China	Both	Age-standardized	Esophageal cancer	Rate	2008	445,10	494,75	395,60
DALYs (Disability-Adjusted Life Years)	China	Both	Age-standardized	Esophageal cancer	Rate	2005	533,01	589,74	461,18
DALYs (Disability-Adjusted Life Years)	Global	Both	Age-standardized	Esophageal cancer	Rate	1997	219,01	234,82	199,06
DALYs (Disability-Adjusted Life Years)	China	Both	Age-standardized	Esophageal cancer	Rate	2006	492,21	545,83	424,63
DALYs (Disability-Adjusted Life Years)	China	Both	Age-standardized	Esophageal cancer	Rate	2014	343,29	395,03	294,41
DALYs (Disability-Adjusted Life Years)	China	Both	Age-standardized	Esophageal cancer	Rate	2013	361,35	409,49	317,44
DALYs (Disability-Adjusted Life Years)	China	Both	Age-standardized	Esophageal cancer	Rate	2017	320,13	384,06	267,22
DALYs (Disability-Adjusted Life Years)	China	Both	Age-standardized	Esophageal cancer	Rate	2012	379,43	431,53	333,75
DALYs (Disability-Adjusted Life Years)	China	Both	Age-standardized	Esophageal cancer	Rate	2016	326,76	382,43	276,12
DALYs (Disability-Adjusted Life Years)	China	Both	Age-standardized	Esophageal cancer	Rate	2015	332,92	383,88	282,08
DALYs (Disability-Adjusted Life Years)	China	Both	Age-standardized	Esophageal cancer	Rate	2011	395,13	443,88	349,36
DALYs (Disability-Adjusted Life Years)	Global	Both	Age-standardized	Esophageal cancer	Rate	2004	211,14	226,75	191,39
DALYs (Disability-Adjusted Life Years)	Global	Both	Age-standardized	Esophageal cancer	Rate	2002	213,38	228,48	191,67
DALYs (Disability-Adjusted Life Years)	Global	Both	Age-standardized	Esophageal cancer	Rate	2007	188,98	201,81	171,50
DALYs (Disability-Adjusted Life Years)	Global	Both	Age-standardized	Esophageal cancer	Rate	2005	205,65	219,40	187,73
DALYs (Disability-Adjusted Life Years)	China	Both	Age-standardized	Esophageal cancer	Rate	2019	318,04	387,10	254,49
DALYs (Disability-Adjusted Life Years)	Global	Both	Age-standardized	Esophageal cancer	Rate	2003	212,35	226,59	191,40
DALYs (Disability-Adjusted Life Years)	China	Both	Age-standardized	Esophageal cancer	Rate	2020	318,34	385,88	253,87
DALYs (Disability-Adjusted Life Years)	China	Both	Age-standardized	Esophageal cancer	Rate	2021	317,18	392,42	252,46
DALYs (Disability-Adjusted Life Years)	Global	Both	Age-standardized	Esophageal cancer	Rate	2006	195,82	208,78	179,76
DALYs (Disability-Adjusted Life Years)	China	Both	Age-standardized	Esophageal cancer	Rate	2018	318,50	381,69	257,85
DALYs (Disability-Adjusted Life Years)	Global	Both	Age-standardized	Esophageal cancer	Rate	2012	165,44	179,22	153,87
DALYs (Disability-Adjusted Life Years)	Global	Both	Age-standardized	Esophageal cancer	Rate	2013	160,52	174,29	148,21
DALYs (Disability-Adjusted Life Years)	Global	Both	Age-standardized	Esophageal cancer	Rate	2011	170,06	182,62	158,17
DALYs (Disability-Adjusted Life Years)	Global	Both	Age-standardized	Esophageal cancer	Rate	2009	178,91	190,44	164,91
DALYs (Disability-Adjusted Life Years)	Global	Both	Age-standardized	Esophageal cancer	Rate	2010	174,78	187,47	160,74
DALYs (Disability-Adjusted Life Years)	Global	Both	Age-standardized	Esophageal cancer	Rate	2008	183,78	195,72	170,42
DALYs (Disability-Adjusted Life Years)	Global	Both	Age-standardized	Esophageal cancer	Rate	2014	156,11	169,46	143,10
DALYs (Disability-Adjusted Life Years)	Global	Both	Age-standardized	Esophageal cancer	Rate	2015	153,87	166,87	140,76
DALYs (Disability-Adjusted Life Years)	Global	Both	Age-standardized	Esophageal cancer	Rate	2016	152,44	166,92	138,30

DALYs (Disability-Adjusted Life Years)	Global	Both	Age-standardized	Esophageal cancer	Rate	2019	149,63	166,93	133,20
DALYs (Disability-Adjusted Life Years)	Global	Both	Age-standardized	Esophageal cancer	Rate	2017	150,27	167,71	136,34
DALYs (Disability-Adjusted Life Years)	Global	Both	Age-standardized	Esophageal cancer	Rate	2018	149,80	166,55	133,99
DALYs (Disability-Adjusted Life Years)	Global	Both	Age-standardized	Esophageal cancer	Rate	2021	148,56	166,82	131,71
DALYs (Disability-Adjusted Life Years)	Global	Both	Age-standardized	Esophageal cancer	Rate	2020	148,87	167,34	132,19
Deaths	Global	Both	All ages	Esophageal cancer	Number	1992	367854	402697	332830
Deaths	Global	Both	All ages	Esophageal cancer	Number	1997	391929	419544	355991
Deaths	Global	Both	All ages	Esophageal cancer	Number	1994	379482	409554	344458
Deaths	Global	Both	All ages	Esophageal cancer	Number	1990	356263	390154	319363
Deaths	China	Both	All ages	Esophageal cancer	Number	1990	210821	244587	176081
Deaths	China	Both	All ages	Esophageal cancer	Number	1992	216089	250526	181479
Deaths	China	Both	All ages	Esophageal cancer	Number	1994	220549	249108	187036
Deaths	China	Both	All ages	Esophageal cancer	Number	1997	226558	253136	193991
Deaths	Global	Both	All ages	Esophageal cancer	Number	1998	398784	424728	355525
Deaths	Global	Both	All ages	Esophageal cancer	Number	1995	385123	412402	343147
Deaths	China	Both	All ages	Esophageal cancer	Number	1999	236386	263482	199594
Deaths	China	Both	All ages	Esophageal cancer	Number	1998	230449	256628	193358
Deaths	Global	Both	All ages	Esophageal cancer	Number	1991	362080	395135	325950
Deaths	China	Both	All ages	Esophageal cancer	Number	1991	213826	246310	180581
Deaths	China	Both	All ages	Esophageal cancer	Number	1995	224144	251784	187216
Deaths	Global	Both	All ages	Esophageal cancer	Number	2003	447142	476139	401775
Deaths	China	Both	All ages	Esophageal cancer	Number	2005	271912	301455	234015
Deaths	China	Both	All ages	Esophageal cancer	Number	2003	265515	295403	224543
Deaths	Global	Both	All ages	Esophageal cancer	Number	2000	419305	451944	377789
Deaths	Global	Both	All ages	Esophageal cancer	Number	2001	428697	461453	385859
Deaths	China	Both	All ages	Esophageal cancer	Number	2000	246171	276556	209511
Deaths	China	Both	All ages	Esophageal cancer	Number	2004	274244	305673	234203
Deaths	Global	Both	All ages	Esophageal cancer	Number	2006	447654	478727	405381
Deaths	China	Both	All ages	Esophageal cancer	Number	2006	259698	288001	222440
Deaths	Global	Both	All ages	Esophageal cancer	Number	1999	406979	435928	364356
Deaths	China	Both	All ages	Esophageal cancer	Number	2001	252972	284784	214759

Deaths	Global	Both	All ages	Esophageal cancer	Number	2004	457473	490359	412003
Deaths	Global	Both	All ages	Esophageal cancer	Number	2008	447050	476379	412161
Deaths	Global	Both	All ages	Esophageal cancer	Number	2005	458085	489196	414887
Deaths	China	Both	All ages	Esophageal cancer	Number	2010	254853	286887	219084
Deaths	China	Both	All ages	Esophageal cancer	Number	2008	253433	281622	224265
Deaths	Global	Both	All ages	Esophageal cancer	Number	2007	445063	475896	401655
Deaths	China	Both	All ages	Esophageal cancer	Number	2007	254231	282263	219017
Deaths	Global	Both	All ages	Esophageal cancer	Number	2011	455915	489344	421914
Deaths	China	Both	All ages	Esophageal cancer	Number	2011	254279	285371	223581
Deaths	China	Both	All ages	Esophageal cancer	Number	2009	254500	282037	223093
Deaths	Global	Both	All ages	Esophageal cancer	Number	2013	457698	493594	421943
Deaths	Global	Both	All ages	Esophageal cancer	Number	2009	450325	480337	412913
Deaths	China	Both	All ages	Esophageal cancer	Number	2013	251325	282430	220439
Deaths	Global	Both	All ages	Esophageal cancer	Number	2015	467148	506901	428427
Deaths	Global	Both	All ages	Esophageal cancer	Number	2012	456959	494630	420835
Deaths	Global	Both	All ages	Esophageal cancer	Number	2010	454406	486899	414664
Deaths	China	Both	All ages	Esophageal cancer	Number	2015	250230	287883	214162
Deaths	China	Both	All ages	Esophageal cancer	Number	2012	253691	288068	222976
Deaths	Global	Both	All ages	Esophageal cancer	Number	2014	459463	499145	420001
Deaths	China	Both	All ages	Esophageal cancer	Number	2017	259691	310559	217233
Deaths	Global	Both	All ages	Esophageal cancer	Number	2018	498914	555770	447209
Deaths	Global	Both	All ages	Esophageal cancer	Number	2016	477679	522921	432278
Deaths	China	Both	All ages	Esophageal cancer	Number	2016	255534	297788	216152
Deaths	China	Both	All ages	Esophageal cancer	Number	2014	248362	284749	214253
Deaths	Global	Both	All ages	Esophageal cancer	Number	2019	514058	575458	457445
Deaths	China	Both	All ages	Esophageal cancer	Number	2018	267707	320221	217433
Deaths	Global	Both	All ages	Esophageal cancer	Number	2017	485412	539606	439356
Deaths	Global	Both	All ages	Esophageal cancer	Number	1993	374159	406279	336992
Deaths	Global	Both	All ages	Esophageal cancer	Number	1996	388183	416104	348668
Deaths	China	Both	All ages	Esophageal cancer	Number	2019	277443	334599	225874
Deaths	Global	Both	All ages	Esophageal cancer	Number	2021	538602	603406	475944

Deaths	Global	Both	All ages	Esophageal cancer	Number	2002	436058	467224	389499
Deaths	China	Both	All ages	Esophageal cancer	Number	2021	296443	362831	236648
Deaths	Global	Both	All ages	Esophageal cancer	Number	2020	526254	588828	466272
Deaths	China	Both	All ages	Esophageal cancer	Number	1993	218368	249528	185875
Deaths	China	Both	All ages	Esophageal cancer	Number	1996	224882	252230	189990
Deaths	China	Both	All ages	Esophageal cancer	Number	2002	257688	288141	218177
Deaths	China	Both	All ages	Esophageal cancer	Number	2020	287520	345144	230847
DALYs (Disability-Adjusted Life Years)	Global	Both	All ages	Esophageal cancer	Number	1990	9753566	10739561	8719319
DALYs (Disability-Adjusted Life Years)	China	Both	All ages	Esophageal cancer	Number	1991	5921058	6838260	5005209
DALYs (Disability-Adjusted Life Years)	China	Both	All ages	Esophageal cancer	Number	1990	5852132	6818927	4841614
DALYs (Disability-Adjusted Life Years)	Global	Both	All ages	Esophageal cancer	Number	1994	10272653	11124557	9374630
DALYs (Disability-Adjusted Life Years)	China	Both	All ages	Esophageal cancer	Number	1996	6097656	6824905	5166994
DALYs (Disability-Adjusted Life Years)	China	Both	All ages	Esophageal cancer	Number	1995	6103767	6886628	5155727
DALYs (Disability-Adjusted Life Years)	Global	Both	All ages	Esophageal cancer	Number	1995	10387112	11154880	9327036
DALYs (Disability-Adjusted Life Years)	China	Both	All ages	Esophageal cancer	Number	1993	6001175	6904144	5149451
DALYs (Disability-Adjusted Life Years)	Global	Both	All ages	Esophageal cancer	Number	1993	10165991	11079425	9180725
DALYs (Disability-Adjusted Life Years)	China	Both	All ages	Esophageal cancer	Number	1994	6033051	6849128	5159261
DALYs (Disability-Adjusted Life Years)	China	Both	All ages	Esophageal cancer	Number	1992	5963477	6901823	4982147
DALYs (Disability-Adjusted Life Years)	China	Both	All ages	Esophageal cancer	Number	1997	6109489	6845649	5256416
DALYs (Disability-Adjusted Life Years)	China	Both	All ages	Esophageal cancer	Number	1998	6177606	6892918	5242213
DALYs (Disability-Adjusted Life Years)	China	Both	All ages	Esophageal cancer	Number	2002	6732801	7511867	5758461
DALYs (Disability-Adjusted Life Years)	Global	Both	All ages	Esophageal cancer	Number	1991	9892078	10821162	8918648
DALYs (Disability-Adjusted Life Years)	Global	Both	All ages	Esophageal cancer	Number	1992	10024767	11007515	9025707
DALYs (Disability-Adjusted Life Years)	China	Both	All ages	Esophageal cancer	Number	2001	6643209	7517326	5690694
DALYs (Disability-Adjusted Life Years)	China	Both	All ages	Esophageal cancer	Number	2003	6871596	7655929	5880604
DALYs (Disability-Adjusted Life Years)	China	Both	All ages	Esophageal cancer	Number	2000	6514560	7305104	5603657
DALYs (Disability-Adjusted Life Years)	China	Both	All ages	Esophageal cancer	Number	2004	7055984	7868778	6134066
DALYs (Disability-Adjusted Life Years)	China	Both	All ages	Esophageal cancer	Number	1999	6305559	7014152	5340391
DALYs (Disability-Adjusted Life Years)	Global	Both	All ages	Esophageal cancer	Number	1996	10437031	11171100	9411901
DALYs (Disability-Adjusted Life Years)	Global	Both	All ages	Esophageal cancer	Number	1999	10819845	11571702	9696277
DALYs (Disability-Adjusted Life Years)	Global	Both	All ages	Esophageal cancer	Number	2001	11276797	12140859	10206712

DALYs (Disability-Adjusted Life Years)	China	Both	All ages	Esophageal cancer	Number	2009	6350458	7020298	5559973
DALYs (Disability-Adjusted Life Years)	China	Both	All ages	Esophageal cancer	Number	2010	6299565	7123617	5472702
DALYs (Disability-Adjusted Life Years)	Global	Both	All ages	Esophageal cancer	Number	1998	10636383	11357377	9575056
DALYs (Disability-Adjusted Life Years)	China	Both	All ages	Esophageal cancer	Number	2007	6497175	7207738	5689209
DALYs (Disability-Adjusted Life Years)	Global	Both	All ages	Esophageal cancer	Number	2000	11092840	11942437	10040581
DALYs (Disability-Adjusted Life Years)	China	Both	All ages	Esophageal cancer	Number	2008	6409388	7144229	5688002
DALYs (Disability-Adjusted Life Years)	China	Both	All ages	Esophageal cancer	Number	2005	6963538	7721599	6078349
DALYs (Disability-Adjusted Life Years)	Global	Both	All ages	Esophageal cancer	Number	1997	10500306	11257458	9548951
DALYs (Disability-Adjusted Life Years)	China	Both	All ages	Esophageal cancer	Number	2006	6665799	7406889	5789458
DALYs (Disability-Adjusted Life Years)	China	Both	All ages	Esophageal cancer	Number	2014	6015922	6934641	5158136
DALYs (Disability-Adjusted Life Years)	China	Both	All ages	Esophageal cancer	Number	2013	6127233	6979757	5360929
DALYs (Disability-Adjusted Life Years)	China	Both	All ages	Esophageal cancer	Number	2017	6174075	7435775	5141197
DALYs (Disability-Adjusted Life Years)	China	Both	All ages	Esophageal cancer	Number	2012	6224972	7094427	5472736
DALYs (Disability-Adjusted Life Years)	China	Both	All ages	Esophageal cancer	Number	2016	6105703	7167879	5177025
DALYs (Disability-Adjusted Life Years)	China	Both	All ages	Esophageal cancer	Number	2015	6023825	6950170	5087638
DALYs (Disability-Adjusted Life Years)	China	Both	All ages	Esophageal cancer	Number	2011	6254531	7044457	5537622
DALYs (Disability-Adjusted Life Years)	Global	Both	All ages	Esophageal cancer	Number	2004	11857561	12742315	10752598
DALYs (Disability-Adjusted Life Years)	Global	Both	All ages	Esophageal cancer	Number	2002	11427308	12236592	10271501
DALYs (Disability-Adjusted Life Years)	Global	Both	All ages	Esophageal cancer	Number	2007	11467266	12241148	10432468
DALYs (Disability-Adjusted Life Years)	Global	Both	All ages	Esophageal cancer	Number	2005	11837684	12623647	10823228
DALYs (Disability-Adjusted Life Years)	China	Both	All ages	Esophageal cancer	Number	2019	6525712	7985203	5192326
DALYs (Disability-Adjusted Life Years)	Global	Both	All ages	Esophageal cancer	Number	2003	11640595	12414635	10499616
DALYs (Disability-Adjusted Life Years)	China	Both	All ages	Esophageal cancer	Number	2020	6725776	8200331	5331600
DALYs (Disability-Adjusted Life Years)	China	Both	All ages	Esophageal cancer	Number	2021	6898666	8553366	5471181
DALYs (Disability-Adjusted Life Years)	Global	Both	All ages	Esophageal cancer	Number	2006	11572919	12327849	10635805
DALYs (Disability-Adjusted Life Years)	China	Both	All ages	Esophageal cancer	Number	2018	6334216	7613820	5124183
DALYs (Disability-Adjusted Life Years)	Global	Both	All ages	Esophageal cancer	Number	2012	11454949	12409070	10648807
DALYs (Disability-Adjusted Life Years)	Global	Both	All ages	Esophageal cancer	Number	2013	11421672	12401925	10548378
DALYs (Disability-Adjusted Life Years)	Global	Both	All ages	Esophageal cancer	Number	2011	11455478	12309224	10661743
DALYs (Disability-Adjusted Life Years)	Global	Both	All ages	Esophageal cancer	Number	2009	11428036	12164406	10553733
DALYs (Disability-Adjusted Life Years)	Global	Both	All ages	Esophageal cancer	Number	2010	11460818	12296418	10552099

DALYs (Disability-Adjusted Life Years)	Global	Both	All ages	Esophageal cancer	Number	2008	11440284	12176300	10635688
DALYs (Disability-Adjusted Life Years)	Global	Both	All ages	Esophageal cancer	Number	2014	11411292	12389263	10464314
DALYs (Disability-Adjusted Life Years)	Global	Both	All ages	Esophageal cancer	Number	2015	11553917	12536984	10567232
DALYs (Disability-Adjusted Life Years)	Global	Both	All ages	Esophageal cancer	Number	2016	11755256	12875245	10677773
DALYs (Disability-Adjusted Life Years)	Global	Both	All ages	Esophageal cancer	Number	2019	12492269	13935620	11121474
DALYs (Disability-Adjusted Life Years)	Global	Both	All ages	Esophageal cancer	Number	2017	11900882	13292874	10789954
DALYs (Disability-Adjusted Life Years)	Global	Both	All ages	Esophageal cancer	Number	2018	12182365	13549710	10882924
DALYs (Disability-Adjusted Life Years)	Global	Both	All ages	Esophageal cancer	Number	2021	12999265	14605268	11522861
DALYs (Disability-Adjusted Life Years)	Global	Both	All ages	Esophageal cancer	Number	2020	12740158	14334726	11297431

measure	location	sex	cause	age	joinpoint	AAPC.Index	Start.Obs	End.Obs
Deaths	China	Both	Esophageal cancer	Age-standardized	5	Full Range	1990	2021
Deaths	Global	Both	Esophageal cancer	Age-standardized	4	Full Range	1990	2021
DALYs (Disability-Adjusted Life Years)	China	Both	Esophageal cancer	Age-standardized	4	Full Range	1990	2021
DALYs (Disability-Adjusted Life Years)	Global	Both	Esophageal cancer	Age-standardized	4	Full Range	1990	2021

measure	location	sex	cause	age	joinpoint	Segment	Segment.Start	Segment.End
Deaths	China	Both	Esophageal cancer	Age-standardized	5	0	1990	1998
Deaths	China	Both	Esophageal cancer	Age-standardized	5	1	1998	2004
Deaths	China	Both	Esophageal cancer	Age-standardized	5	2	2004	2007
Deaths	China	Both	Esophageal cancer	Age-standardized	5	3	2007	2010
Deaths	China	Both	Esophageal cancer	Age-standardized	5	4	2010	2015
Deaths	China	Both	Esophageal cancer	Age-standardized	5	5	2015	2021
Deaths	Global	Both	Esophageal cancer	Age-standardized	4	0	1990	1998
Deaths	Global	Both	Esophageal cancer	Age-standardized	4	1	1998	2004
Deaths	Global	Both	Esophageal cancer	Age-standardized	4	2	2004	2007
Deaths	Global	Both	Esophageal cancer	Age-standardized	4	3	2007	2015

Deaths	Global	Both	Esophageal cancer	Age-standardized	4	4	2015	2021
DALYs (Disability-Adjusted Life Years)	China	Both	Esophageal cancer	Age-standardized	4	0	1990	1998
DALYs (Disability-Adjusted Life Years)	China	Both	Esophageal cancer	Age-standardized	4	1	1998	2004
DALYs (Disability-Adjusted Life Years)	China	Both	Esophageal cancer	Age-standardized	4	2	2004	2007
DALYs (Disability-Adjusted Life Years)	China	Both	Esophageal cancer	Age-standardized	4	3	2007	2016
DALYs (Disability-Adjusted Life Years)	China	Both	Esophageal cancer	Age-standardized	4	4	2016	2021
DALYs (Disability-Adjusted Life Years)	Global	Both	Esophageal cancer	Age-standardized	4	0	1990	1998
DALYs (Disability-Adjusted Life Years)	Global	Both	Esophageal cancer	Age-standardized	4	1	1998	2004
DALYs (Disability-Adjusted Life Years)	Global	Both	Esophageal cancer	Age-standardized	4	2	2004	2007
DALYs (Disability-Adjusted Life Years)	Global	Both	Esophageal cancer	Age-standardized	4	3	2007	2015
DALYs (Disability-Adjusted Life Years)	Global	Both	Esophageal cancer	Age-standardized	4	4	2015	2021

AAPC_95CI	Significant_indicator	Test.Statistic	P.Value
-1.98 (-2.19 to -1.77)	Yes	-18,6571	0
-1.2 (-1.33 to -1.07)	Yes	-18,113	0
-2.31 (-2.46 to -2.16)	Yes	-29,3746	0
-1.5 (-1.61 to -1.39)	Yes	-26,4618	0

APC_95CI	Significant_indicator	Test.Statistic	P.Value
-1.42 (-1.6 to -1.25)	Yes	-17,4108	0
0.49 (0.15 to 0.82)	Yes	3,0649	0,008
-5.87 (-7.23 to -4.48)	Yes	-8,8177	0
-3.06 (-4.33 to -1.77)	Yes	-5,0199	0
-4.21 (-4.64 to -3.78)	Yes	-20,3779	0
-0.74 (-1.08 to -0.41)	Yes	-4,6754	0
-0.76 (-0.89 to -0.63)	Yes	-11,964	0
-0.01 (-0.28 to 0.25)	No	-0,0977	0,923
-3.53 (-4.62 to -2.44)	Yes	-6,6859	0
-2.26 (-2.4 to -2.11)	Yes	-33,1717	0

-0.35<br>(-0.6 to -0.1)	Yes	-2,9415	0,009
-1.75<br>(-1.91 to -1.58)	Yes	-22,2957	0
-0.27<br>(-0.57 to 0.04)	No	-1,8294	0,084
-5.99<br>(-7.17 to -4.79)	Yes	-10,2245	0
-4.03<br>(-4.17 to -3.89)	Yes	-58,7824	0
-0.24<br>(-0.71 to 0.23)	No	-1,0897	0,29
-1.02<br>(-1.15 to -0.9)	Yes	-17,4691	0
-0.45<br>(-0.68 to -0.21)	Yes	-4,017	0,001
-3.77<br>(-4.67 to -2.86)	Yes	-8,574	0
-2.62<br>(-2.74 to -2.5)	Yes	-43,9329	0
-0.52<br>(-0.74 to -0.3)	Yes	-4,8538	0

measure	location	sex	age	cause	metric	year	val	upper	lower
Deaths	Global	Both	20-24 years	Esophageal cancer	Rate	1990	0,12	0,13	0,10
Deaths	Global	Both	25-29 years	Esophageal cancer	Rate	1990	0,19	0,21	0,17
Deaths	Global	Both	30-34 years	Esophageal cancer	Rate	1990	0,46	0,51	0,41
Deaths	Global	Both	35-39 years	Esophageal cancer	Rate	1990	1,38	1,57	1,21
Deaths	Global	Both	40-44 years	Esophageal cancer	Rate	1990	3,79	4,33	3,32
Deaths	Global	Both	45-49 years	Esophageal cancer	Rate	1990	7,63	8,54	6,84
Deaths	Global	Both	50-54 years	Esophageal cancer	Rate	1990	15,55	17,34	13,81
Deaths	Global	Both	55-59 years	Esophageal cancer	Rate	1990	25,75	28,84	22,82
Deaths	Global	Both	60-64 years	Esophageal cancer	Rate	1990	35,20	39,13	31,10
Deaths	Global	Both	65-69 years	Esophageal cancer	Rate	1990	46,20	51,17	40,01
Deaths	Global	Both	70-74 years	Esophageal cancer	Rate	1990	60,83	67,32	52,98
Deaths	Global	Both	75-79 years	Esophageal cancer	Rate	1990	62,46	68,42	55,60
Deaths	Global	Both	80-84 years	Esophageal cancer	Rate	1990	61,67	66,63	55,06
Deaths	Global	Both	85-89 years	Esophageal cancer	Rate	1990	67,27	72,69	58,51
Deaths	Global	Both	90-94 years	Esophageal cancer	Rate	1990	64,82	70,17	54,93
Deaths	Global	Both	95+ years	Esophageal cancer	Rate	1990	49,07	55,09	38,63
Deaths	China	Both	20-24 years	Esophageal cancer	Rate	1990	0,23	0,27	0,19
Deaths	China	Both	25-29 years	Esophageal cancer	Rate	1990	0,36	0,42	0,31
Deaths	China	Both	30-34 years	Esophageal cancer	Rate	1990	1,13	1,33	0,97
Deaths	China	Both	35-39 years	Esophageal cancer	Rate	1990	3,53	4,23	2,94
Deaths	China	Both	40-44 years	Esophageal cancer	Rate	1990	10,62	12,87	8,65
Deaths	China	Both	45-49 years	Esophageal cancer	Rate	1990	19,95	23,99	16,39
Deaths	China	Both	50-54 years	Esophageal cancer	Rate	1990	40,42	48,19	33,43
Deaths	China	Both	55-59 years	Esophageal cancer	Rate	1990	66,91	80,17	55,01
Deaths	China	Both	60-64 years	Esophageal cancer	Rate	1990	95,57	113,72	77,39
Deaths	China	Both	65-69 years	Esophageal cancer	Rate	1990	129,37	151,22	103,69
Deaths	China	Both	70-74 years	Esophageal cancer	Rate	1990	176,52	204,32	143,38
Deaths	China	Both	75-79 years	Esophageal cancer	Rate	1990	194,78	223,31	157,63
Deaths	China	Both	80-84 years	Esophageal cancer	Rate	1990	200,21	228,40	166,44
Deaths	China	Both	85-89 years	Esophageal cancer	Rate	1990	245,91	276,95	197,80

Deaths	China	Both	90-94 years	Esophageal cancer	Rate	1990	285,64	324,95	224,64
Deaths	China	Both	95+ years	Esophageal cancer	Rate	1990	211,06	258,25	134,84
Deaths	China	Both	20-24 years	Esophageal cancer	Rate	2021	0,12	0,16	0,10
Deaths	China	Both	25-29 years	Esophageal cancer	Rate	2021	0,17	0,21	0,14
Deaths	China	Both	30-34 years	Esophageal cancer	Rate	2021	0,49	0,59	0,39
Deaths	China	Both	35-39 years	Esophageal cancer	Rate	2021	1,21	1,53	0,95
Deaths	China	Both	40-44 years	Esophageal cancer	Rate	2021	3,46	4,37	2,67
Deaths	China	Both	45-49 years	Esophageal cancer	Rate	2021	7,01	9,15	5,29
Deaths	China	Both	50-54 years	Esophageal cancer	Rate	2021	15,62	19,92	11,90
Deaths	China	Both	55-59 years	Esophageal cancer	Rate	2021	26,70	34,62	20,35
Deaths	China	Both	60-64 years	Esophageal cancer	Rate	2021	43,92	56,36	34,38
Deaths	China	Both	65-69 years	Esophageal cancer	Rate	2021	65,08	80,40	50,60
Deaths	China	Both	70-74 years	Esophageal cancer	Rate	2021	99,12	121,99	79,14
Deaths	China	Both	75-79 years	Esophageal cancer	Rate	2021	129,13	156,91	102,56
Deaths	China	Both	80-84 years	Esophageal cancer	Rate	2021	155,48	186,54	126,54
Deaths	China	Both	85-89 years	Esophageal cancer	Rate	2021	207,58	244,24	169,35
Deaths	China	Both	90-94 years	Esophageal cancer	Rate	2021	207,92	246,09	160,79
Deaths	China	Both	95+ years	Esophageal cancer	Rate	2021	155,42	196,35	103,89
Deaths	Global	Both	20-24 years	Esophageal cancer	Rate	2021	0,07	0,08	0,06
Deaths	Global	Both	25-29 years	Esophageal cancer	Rate	2021	0,12	0,14	0,11
Deaths	Global	Both	30-34 years	Esophageal cancer	Rate	2021	0,27	0,30	0,24
Deaths	Global	Both	35-39 years	Esophageal cancer	Rate	2021	0,63	0,70	0,57
Deaths	Global	Both	40-44 years	Esophageal cancer	Rate	2021	1,68	1,87	1,50
Deaths	Global	Both	45-49 years	Esophageal cancer	Rate	2021	3,86	4,41	3,40
Deaths	Global	Both	50-54 years	Esophageal cancer	Rate	2021	8,49	9,75	7,40
Deaths	Global	Both	55-59 years	Esophageal cancer	Rate	2021	14,40	16,62	12,50
Deaths	Global	Both	60-64 years	Esophageal cancer	Rate	2021	20,37	23,21	18,06
Deaths	Global	Both	65-69 years	Esophageal cancer	Rate	2021	30,97	35,33	26,90
Deaths	Global	Both	70-74 years	Esophageal cancer	Rate	2021	42,48	48,24	37,08
Deaths	Global	Both	75-79 years	Esophageal cancer	Rate	2021	53,20	60,47	46,28
Deaths	Global	Both	80-84 years	Esophageal cancer	Rate	2021	60,16	67,87	51,81

Deaths	Global	Both	85-89 years	Esophageal cancer	Rate	2021	74,51	83,57	63,50
Deaths	Global	Both	90-94 years	Esophageal cancer	Rate	2021	71,45	80,35	58,95
Deaths	Global	Both	95+ years	Esophageal cancer	Rate	2021	57,70	66,19	43,00
DALYs (Disability-Adjusted Life Years)	Global	Both	20-24 years	Esophageal cancer	Rate	1990	7,93	8,82	6,83
DALYs (Disability-Adjusted Life Years)	Global	Both	25-29 years	Esophageal cancer	Rate	1990	12,16	13,21	10,69
DALYs (Disability-Adjusted Life Years)	Global	Both	30-34 years	Esophageal cancer	Rate	1990	26,82	29,64	24,06
DALYs (Disability-Adjusted Life Years)	Global	Both	35-39 years	Esophageal cancer	Rate	1990	73,46	83,63	64,35
DALYs (Disability-Adjusted Life Years)	Global	Both	40-44 years	Esophageal cancer	Rate	1990	183,12	208,93	160,20
DALYs (Disability-Adjusted Life Years)	Global	Both	45-49 years	Esophageal cancer	Rate	1990	330,08	369,05	296,04
DALYs (Disability-Adjusted Life Years)	Global	Both	50-54 years	Esophageal cancer	Rate	1990	598,42	667,26	531,27
DALYs (Disability-Adjusted Life Years)	Global	Both	55-59 years	Esophageal cancer	Rate	1990	870,34	974,11	771,21
DALYs (Disability-Adjusted Life Years)	Global	Both	60-64 years	Esophageal cancer	Rate	1990	1026,16	1140,95	906,93
DALYs (Disability-Adjusted Life Years)	Global	Both	65-69 years	Esophageal cancer	Rate	1990	1134,65	1257,55	984,77
DALYs (Disability-Adjusted Life Years)	Global	Both	70-74 years	Esophageal cancer	Rate	1990	1229,84	1363,05	1072,34
DALYs (Disability-Adjusted Life Years)	Global	Both	75-79 years	Esophageal cancer	Rate	1990	1012,52	1109,06	900,44
DALYs (Disability-Adjusted Life Years)	Global	Both	80-84 years	Esophageal cancer	Rate	1990	783,57	847,84	700,68
DALYs (Disability-Adjusted Life Years)	Global	Both	85-89 years	Esophageal cancer	Rate	1990	680,62	735,78	592,68
DALYs (Disability-Adjusted Life Years)	Global	Both	90-94 years	Esophageal cancer	Rate	1990	568,59	615,89	482,92
DALYs (Disability-Adjusted Life Years)	Global	Both	95+ years	Esophageal cancer	Rate	1990	406,50	455,92	320,69
DALYs (Disability-Adjusted Life Years)	China	Both	20-24 years	Esophageal cancer	Rate	1990	15,83	18,51	13,10
DALYs (Disability-Adjusted Life Years)	China	Both	25-29 years	Esophageal cancer	Rate	1990	22,80	26,36	19,29
DALYs (Disability-Adjusted Life Years)	China	Both	30-34 years	Esophageal cancer	Rate	1990	65,61	76,80	56,15
DALYs (Disability-Adjusted Life Years)	China	Both	35-39 years	Esophageal cancer	Rate	1990	187,80	225,22	156,04
DALYs (Disability-Adjusted Life Years)	China	Both	40-44 years	Esophageal cancer	Rate	1990	512,55	621,61	417,38
DALYs (Disability-Adjusted Life Years)	China	Both	45-49 years	Esophageal cancer	Rate	1990	863,21	1038,08	709,08
DALYs (Disability-Adjusted Life Years)	China	Both	50-54 years	Esophageal cancer	Rate	1990	1553,31	1853,96	1283,32
DALYs (Disability-Adjusted Life Years)	China	Both	55-59 years	Esophageal cancer	Rate	1990	2261,81	2713,09	1860,12
DALYs (Disability-Adjusted Life Years)	China	Both	60-64 years	Esophageal cancer	Rate	1990	2784,86	3310,28	2255,36
DALYs (Disability-Adjusted Life Years)	China	Both	65-69 years	Esophageal cancer	Rate	1990	3172,78	3711,12	2542,15
DALYs (Disability-Adjusted Life Years)	China	Both	70-74 years	Esophageal cancer	Rate	1990	3564,81	4132,25	2892,26
DALYs (Disability-Adjusted Life Years)	China	Both	75-79 years	Esophageal cancer	Rate	1990	3160,24	3629,64	2559,24

DALYs (Disability-Adjusted Life Years)	China	Both	80-84 years	Esophageal cancer	Rate	1990	2542,31	2900,74	2117,07
DALYs (Disability-Adjusted Life Years)	China	Both	85-89 years	Esophageal cancer	Rate	1990	2491,23	2810,15	2003,84
DALYs (Disability-Adjusted Life Years)	China	Both	90-94 years	Esophageal cancer	Rate	1990	2507,43	2849,70	1973,35
DALYs (Disability-Adjusted Life Years)	China	Both	95+ years	Esophageal cancer	Rate	1990	1766,51	2161,55	1127,94
DALYs (Disability-Adjusted Life Years)	China	Both	20-24 years	Esophageal cancer	Rate	2021	8,28	10,62	6,51
DALYs (Disability-Adjusted Life Years)	China	Both	25-29 years	Esophageal cancer	Rate	2021	10,85	13,06	8,76
DALYs (Disability-Adjusted Life Years)	China	Both	30-34 years	Esophageal cancer	Rate	2021	28,25	34,37	22,79
DALYs (Disability-Adjusted Life Years)	China	Both	35-39 years	Esophageal cancer	Rate	2021	64,62	81,61	50,88
DALYs (Disability-Adjusted Life Years)	China	Both	40-44 years	Esophageal cancer	Rate	2021	167,17	211,75	128,80
DALYs (Disability-Adjusted Life Years)	China	Both	45-49 years	Esophageal cancer	Rate	2021	303,31	396,49	229,50
DALYs (Disability-Adjusted Life Years)	China	Both	50-54 years	Esophageal cancer	Rate	2021	601,60	766,41	458,38
DALYs (Disability-Adjusted Life Years)	China	Both	55-59 years	Esophageal cancer	Rate	2021	905,61	1173,72	690,37
DALYs (Disability-Adjusted Life Years)	China	Both	60-64 years	Esophageal cancer	Rate	2021	1278,85	1641,81	1001,59
DALYs (Disability-Adjusted Life Years)	China	Both	65-69 years	Esophageal cancer	Rate	2021	1597,51	1971,30	1240,94
DALYs (Disability-Adjusted Life Years)	China	Both	70-74 years	Esophageal cancer	Rate	2021	2004,99	2462,56	1601,00
DALYs (Disability-Adjusted Life Years)	China	Both	75-79 years	Esophageal cancer	Rate	2021	2091,81	2545,53	1664,98
DALYs (Disability-Adjusted Life Years)	China	Both	80-84 years	Esophageal cancer	Rate	2021	1966,07	2360,14	1596,11
DALYs (Disability-Adjusted Life Years)	China	Both	85-89 years	Esophageal cancer	Rate	2021	2088,89	2459,16	1706,44
DALYs (Disability-Adjusted Life Years)	China	Both	90-94 years	Esophageal cancer	Rate	2021	1823,55	2157,37	1414,86
DALYs (Disability-Adjusted Life Years)	China	Both	95+ years	Esophageal cancer	Rate	2021	1288,84	1629,15	862,86
DALYs (Disability-Adjusted Life Years)	Global	Both	20-24 years	Esophageal cancer	Rate	2021	4,68	5,56	4,15
DALYs (Disability-Adjusted Life Years)	Global	Both	25-29 years	Esophageal cancer	Rate	2021	7,43	8,59	6,65
DALYs (Disability-Adjusted Life Years)	Global	Both	30-34 years	Esophageal cancer	Rate	2021	15,76	17,50	14,25
DALYs (Disability-Adjusted Life Years)	Global	Both	35-39 years	Esophageal cancer	Rate	2021	33,55	37,17	30,12
DALYs (Disability-Adjusted Life Years)	Global	Both	40-44 years	Esophageal cancer	Rate	2021	81,06	90,20	72,26
DALYs (Disability-Adjusted Life Years)	Global	Both	45-49 years	Esophageal cancer	Rate	2021	167,12	190,88	147,41
DALYs (Disability-Adjusted Life Years)	Global	Both	50-54 years	Esophageal cancer	Rate	2021	327,29	375,73	285,85
DALYs (Disability-Adjusted Life Years)	Global	Both	55-59 years	Esophageal cancer	Rate	2021	488,21	564,35	423,61
DALYs (Disability-Adjusted Life Years)	Global	Both	60-64 years	Esophageal cancer	Rate	2021	594,12	677,76	527,23
DALYs (Disability-Adjusted Life Years)	Global	Both	65-69 years	Esophageal cancer	Rate	2021	761,44	867,27	660,90
DALYs (Disability-Adjusted Life Years)	Global	Both	70-74 years	Esophageal cancer	Rate	2021	860,29	979,26	752,09

DALYs (Disability-Adjusted Life Years)	Global	Both	75-79 years	Esophageal cancer	Rate	2021	862,33	979,69	751,32
DALYs (Disability-Adjusted Life Years)	Global	Both	80-84 years	Esophageal cancer	Rate	2021	762,55	860,31	657,14
DALYs (Disability-Adjusted Life Years)	Global	Both	85-89 years	Esophageal cancer	Rate	2021	751,01	842,61	640,57
DALYs (Disability-Adjusted Life Years)	Global	Both	90-94 years	Esophageal cancer	Rate	2021	626,91	705,01	516,10
DALYs (Disability-Adjusted Life Years)	Global	Both	95+ years	Esophageal cancer	Rate	2021	473,96	544,82	353,48

measure	location	sex	age	cause	metric	year	pred_val	pred_low	pred_up
Deaths	China	Both	All ages	Esophageal cancer	Number	2022	311501	273777	349225
Deaths	China	Both	All ages	Esophageal cancer	Number	2023	321523	277297	365748
Deaths	China	Both	All ages	Esophageal cancer	Number	2024	332218	277838	386599
Deaths	China	Both	All ages	Esophageal cancer	Number	2025	343350	275156	411544
Deaths	China	Both	All ages	Esophageal cancer	Number	2026	354628	269142	440114
Deaths	China	Both	All ages	Esophageal cancer	Number	2027	365718	259667	471770
Deaths	China	Both	All ages	Esophageal cancer	Number	2028	376986	247081	506892
Deaths	China	Both	All ages	Esophageal cancer	Number	2029	389014	231669	546359
Deaths	China	Both	All ages	Esophageal cancer	Number	2030	401796	213154	590437
Deaths	China	Both	All ages	Esophageal cancer	Number	2031	415240	191113	639367
Deaths	China	Both	All ages	Esophageal cancer	Number	2032	429147	165018	693276
Deaths	China	Both	All ages	Esophageal cancer	Number	2033	444064	134574	753558
Deaths	China	Both	All ages	Esophageal cancer	Number	2034	460602	99182	822031
Deaths	China	Both	All ages	Esophageal cancer	Number	2035	478673	64279	899610
Deaths	Global	Both	All ages	Esophageal cancer	Number	2022	560163	522743	597582
Deaths	Global	Both	All ages	Esophageal cancer	Number	2023	574352	531035	617668
Deaths	Global	Both	All ages	Esophageal cancer	Number	2024	589343	535563	643122
Deaths	Global	Both	All ages	Esophageal cancer	Number	2025	604734	537036	672432
Deaths	Global	Both	All ages	Esophageal cancer	Number	2026	620412	535279	705546
Deaths	Global	Both	All ages	Esophageal cancer	Number	2027	635913	530048	741777
Deaths	Global	Both	All ages	Esophageal cancer	Number	2028	651433	521945	780920
Deaths	Global	Both	All ages	Esophageal cancer	Number	2029	667436	511425	823447
Deaths	Global	Both	All ages	Esophageal cancer	Number	2030	683976	498452	869500
Deaths	Global	Both	All ages	Esophageal cancer	Number	2031	701135	482886	919384
Deaths	Global	Both	All ages	Esophageal cancer	Number	2032	718532	464209	972855
Deaths	Global	Both	All ages	Esophageal cancer	Number	2033	736408	442592	1030223
Deaths	Global	Both	All ages	Esophageal cancer	Number	2034	755138	418105	1092171
Deaths	Global	Both	All ages	Esophageal cancer	Number	2035	774676	390422	1158930
DALYs (Disability-Adjusted Life Years)	China	Both	All ages	Esophageal cancer	Number	2022	7039697	6052321	8027073
DALYs (Disability-Adjusted Life Years)	China	Both	All ages	Esophageal cancer	Number	2023	7197615	6084066	8311163
DALYs (Disability-Adjusted Life Years)	China	Both	All ages	Esophageal cancer	Number	2024	7361030	6050097	8671962

DALYs (Disability-Adjusted Life Years)	China	Both	All ages	Esophageal cancer	Number	2025	7527455	5946485	9108425
DALYs (Disability-Adjusted Life Years)	China	Both	All ages	Esophageal cancer	Number	2026	7693885	5773725	9614044
DALYs (Disability-Adjusted Life Years)	China	Both	All ages	Esophageal cancer	Number	2027	7855265	5531996	10178534
DALYs (Disability-Adjusted Life Years)	China	Both	All ages	Esophageal cancer	Number	2028	8015045	5227288	10802803
DALYs (Disability-Adjusted Life Years)	China	Both	All ages	Esophageal cancer	Number	2029	8181245	4865463	11497027
DALYs (Disability-Adjusted Life Years)	China	Both	All ages	Esophageal cancer	Number	2030	8355386	4444959	12265814
DALYs (Disability-Adjusted Life Years)	China	Both	All ages	Esophageal cancer	Number	2031	8538860	3961814	13115907
DALYs (Disability-Adjusted Life Years)	China	Both	All ages	Esophageal cancer	Number	2032	8730869	3409184	14052554
DALYs (Disability-Adjusted Life Years)	China	Both	All ages	Esophageal cancer	Number	2033	8938327	2781597	15095058
DALYs (Disability-Adjusted Life Years)	China	Both	All ages	Esophageal cancer	Number	2034	9168324	2068930	16267866
DALYs (Disability-Adjusted Life Years)	China	Both	All ages	Esophageal cancer	Number	2035	9420344	1440762	17586902
DALYs (Disability-Adjusted Life Years)	Global	Both	All ages	Esophageal cancer	Number	2022	13321007	12390692	14251322
DALYs (Disability-Adjusted Life Years)	Global	Both	All ages	Esophageal cancer	Number	2023	13585444	12509542	14661345
DALYs (Disability-Adjusted Life Years)	Global	Both	All ages	Esophageal cancer	Number	2024	13849173	12545215	15153130
DALYs (Disability-Adjusted Life Years)	Global	Both	All ages	Esophageal cancer	Number	2025	14110611	12498954	15722268
DALYs (Disability-Adjusted Life Years)	Global	Both	All ages	Esophageal cancer	Number	2026	14376958	12382920	16370996
DALYs (Disability-Adjusted Life Years)	Global	Both	All ages	Esophageal cancer	Number	2027	14644546	12198946	17090147
DALYs (Disability-Adjusted Life Years)	Global	Both	All ages	Esophageal cancer	Number	2028	14913105	11954437	17871773
DALYs (Disability-Adjusted Life Years)	Global	Both	All ages	Esophageal cancer	Number	2029	15181689	11651977	18711400
DALYs (Disability-Adjusted Life Years)	Global	Both	All ages	Esophageal cancer	Number	2030	15448143	11290652	19605633
DALYs (Disability-Adjusted Life Years)	Global	Both	All ages	Esophageal cancer	Number	2031	15724986	10877736	20572235
DALYs (Disability-Adjusted Life Years)	Global	Both	All ages	Esophageal cancer	Number	2032	16015233	10411532	21618935
DALYs (Disability-Adjusted Life Years)	Global	Both	All ages	Esophageal cancer	Number	2033	16321320	9892958	22749682
DALYs (Disability-Adjusted Life Years)	Global	Both	All ages	Esophageal cancer	Number	2034	16638141	9315703	23960580
DALYs (Disability-Adjusted Life Years)	Global	Both	All ages	Esophageal cancer	Number	2035	16958312	8671143	25245481
Deaths	China	Both	Age-standardized	Esophageal cancer	Rate	2022	14,48	12,72	16,24
Deaths	China	Both	Age-standardized	Esophageal cancer	Rate	2023	14,49	12,49	16,49
Deaths	China	Both	Age-standardized	Esophageal cancer	Rate	2024	14,49	12,12	16,87
Deaths	China	Both	Age-standardized	Esophageal cancer	Rate	2025	14,50	11,62	17,39
Deaths	China	Both	Age-standardized	Esophageal cancer	Rate	2026	14,51	11,01	18,02
Deaths	China	Both	Age-standardized	Esophageal cancer	Rate	2027	14,52	10,31	18,74
Deaths	China	Both	Age-standardized	Esophageal cancer	Rate	2028	14,54	9,53	19,56

Deaths	China	Both	Age-standardized	Esophageal cancer	Rate	2029	14,58	8,68	20,48
Deaths	China	Both	Age-standardized	Esophageal cancer	Rate	2030	14,63	7,75	21,50
Deaths	China	Both	Age-standardized	Esophageal cancer	Rate	2031	14,69	6,76	22,62
Deaths	China	Both	Age-standardized	Esophageal cancer	Rate	2032	14,78	5,68	23,87
Deaths	China	Both	Age-standardized	Esophageal cancer	Rate	2033	14,89	4,51	25,26
Deaths	China	Both	Age-standardized	Esophageal cancer	Rate	2034	15,02	3,24	26,81
Deaths	China	Both	Age-standardized	Esophageal cancer	Rate	2035	15,20	2,05	28,55
Deaths	Global	Both	Age-standardized	Esophageal cancer	Rate	2022	6,27	5,85	6,69
Deaths	Global	Both	Age-standardized	Esophageal cancer	Rate	2023	6,26	5,78	6,73
Deaths	Global	Both	Age-standardized	Esophageal cancer	Rate	2024	6,24	5,67	6,81
Deaths	Global	Both	Age-standardized	Esophageal cancer	Rate	2025	6,23	5,53	6,93
Deaths	Global	Both	Age-standardized	Esophageal cancer	Rate	2026	6,22	5,36	7,07
Deaths	Global	Both	Age-standardized	Esophageal cancer	Rate	2027	6,20	5,17	7,24
Deaths	Global	Both	Age-standardized	Esophageal cancer	Rate	2028	6,19	4,96	7,42
Deaths	Global	Both	Age-standardized	Esophageal cancer	Rate	2029	6,18	4,73	7,62
Deaths	Global	Both	Age-standardized	Esophageal cancer	Rate	2030	6,16	4,49	7,83
Deaths	Global	Both	Age-standardized	Esophageal cancer	Rate	2031	6,15	4,24	8,07
Deaths	Global	Both	Age-standardized	Esophageal cancer	Rate	2032	6,15	3,97	8,32
Deaths	Global	Both	Age-standardized	Esophageal cancer	Rate	2033	6,15	3,69	8,60
Deaths	Global	Both	Age-standardized	Esophageal cancer	Rate	2034	6,15	3,40	8,89
Deaths	Global	Both	Age-standardized	Esophageal cancer	Rate	2035	6,15	3,10	9,20
DALYs (Disability-Adjusted Life Years)	China	Both	Age-standardized	Esophageal cancer	Rate	2022	314,81	270,64	358,99
DALYs (Disability-Adjusted Life Years)	China	Both	Age-standardized	Esophageal cancer	Rate	2023	313,47	264,94	362,00
DALYs (Disability-Adjusted Life Years)	China	Both	Age-standardized	Esophageal cancer	Rate	2024	312,19	256,55	367,83
DALYs (Disability-Adjusted Life Years)	China	Both	Age-standardized	Esophageal cancer	Rate	2025	310,99	245,62	376,35
DALYs (Disability-Adjusted Life Years)	China	Both	Age-standardized	Esophageal cancer	Rate	2026	309,95	232,54	387,35
DALYs (Disability-Adjusted Life Years)	China	Both	Age-standardized	Esophageal cancer	Rate	2027	309,12	217,65	400,59
DALYs (Disability-Adjusted Life Years)	China	Both	Age-standardized	Esophageal cancer	Rate	2028	308,60	201,23	415,96
DALYs (Disability-Adjusted Life Years)	China	Both	Age-standardized	Esophageal cancer	Rate	2029	308,43	183,43	433,43
DALYs (Disability-Adjusted Life Years)	China	Both	Age-standardized	Esophageal cancer	Rate	2030	308,65	164,24	453,06
DALYs (Disability-Adjusted Life Years)	China	Both	Age-standardized	Esophageal cancer	Rate	2031	309,36	143,63	475,09
DALYs (Disability-Adjusted Life Years)	China	Both	Age-standardized	Esophageal cancer	Rate	2032	310,66	121,47	499,86

DALYs (Disability-Adjusted Life Years)	China	Both	Age-standardized	Esophageal cancer	Rate	2033	312,67	97,55	527,78
DALYs (Disability-Adjusted Life Years)	China	Both	Age-standardized	Esophageal cancer	Rate	2034	315,42	71,55	559,30
DALYs (Disability-Adjusted Life Years)	China	Both	Age-standardized	Esophageal cancer	Rate	2035	318,95	49,03	594,94
DALYs (Disability-Adjusted Life Years)	Global	Both	Age-standardized	Esophageal cancer	Rate	2022	147,28	137,00	157,57
DALYs (Disability-Adjusted Life Years)	Global	Both	Age-standardized	Esophageal cancer	Rate	2023	146,60	134,99	158,22
DALYs (Disability-Adjusted Life Years)	Global	Both	Age-standardized	Esophageal cancer	Rate	2024	145,88	132,14	159,61
DALYs (Disability-Adjusted Life Years)	Global	Both	Age-standardized	Esophageal cancer	Rate	2025	145,08	128,51	161,66
DALYs (Disability-Adjusted Life Years)	Global	Both	Age-standardized	Esophageal cancer	Rate	2026	144,34	124,31	164,37
DALYs (Disability-Adjusted Life Years)	Global	Both	Age-standardized	Esophageal cancer	Rate	2027	143,68	119,68	167,68
DALYs (Disability-Adjusted Life Years)	Global	Both	Age-standardized	Esophageal cancer	Rate	2028	143,09	114,69	171,50
DALYs (Disability-Adjusted Life Years)	Global	Both	Age-standardized	Esophageal cancer	Rate	2029	142,51	109,36	175,66
DALYs (Disability-Adjusted Life Years)	Global	Both	Age-standardized	Esophageal cancer	Rate	2030	141,90	103,69	180,11
DALYs (Disability-Adjusted Life Years)	Global	Both	Age-standardized	Esophageal cancer	Rate	2031	141,38	97,78	184,99
DALYs (Disability-Adjusted Life Years)	Global	Both	Age-standardized	Esophageal cancer	Rate	2032	141,04	91,66	190,43
DALYs (Disability-Adjusted Life Years)	Global	Both	Age-standardized	Esophageal cancer	Rate	2033	140,90	85,36	196,43
DALYs (Disability-Adjusted Life Years)	Global	Both	Age-standardized	Esophageal cancer	Rate	2034	140,84	78,81	202,88
DALYs (Disability-Adjusted Life Years)	Global	Both	Age-standardized	Esophageal cancer	Rate	2035	140,79	71,93	209,66

©2024 Wang M. and Miao H.
